# Supplementary material for: Mechanisms of Cellular Responses of the Natural Alkaloid Caulerpin and Its Similarities with the Lipid-Lowering Agent Fenofibrate in Mytilus galloprovincialis
Source: Toxins (Basel). 2025 Oct 18;17(10):512. doi: 10.3390/toxins17100512 (PMC12567801; doi:10.3390/toxins17100512)
Supplement: Supplementary file 1 [file toxins-17-00512-s001.zip › toxins-3728018-supplementary.pdf]

*Article*

# Supplementary Material: Mechanisms of Cellular Responses of the Natural Alkaloid Caulerpin and Its Similarities with the Lipid-Lowering Agent Fenofibrate in *Mytilus galloprovincialis*

**Michela Panni, Marica Mezzelani, Maria Elisa Giuliani, Paola Nisi Cerioni, Alessandro Nardi, Ernesto Mollo, Francesco Regoli, Maura Benedetti, and Stefania Gorbi**

Supplementary material

## Selected chemicals

Fenofibrate (FFB, CAS 49562-28-9) was purchased from Sigma-Aldrich® (St. Louis, MO, USA). Caulerpin (CAU) was extracted and purified from *Caulerpa cylindracea* following the procedure described by Vitale et al. (2019). An initial stock solution (5 mM) of the purified compound was kindly provided by the Institute of Biomolecular Chemistry (National Research Council, Italy). The chemical structures of CAU and FFB are reported in Figure S1.

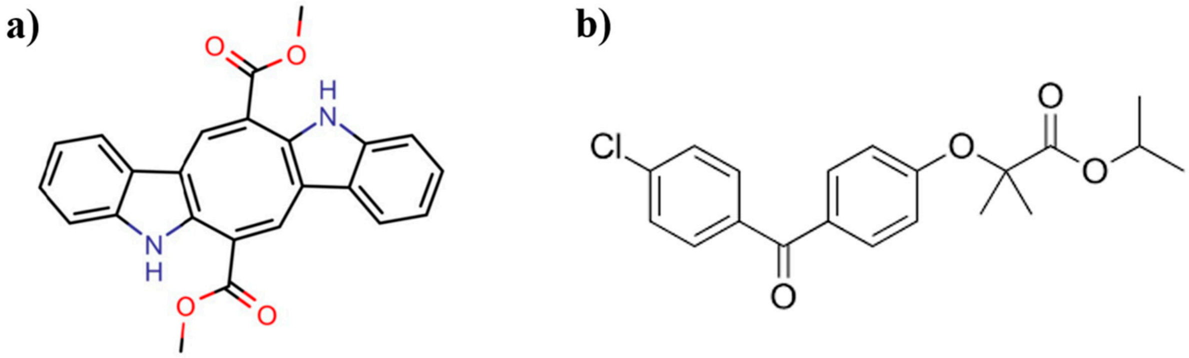


**Figure S1.** Chemical structures of a) caulerpin and b) fenofibrate.

**Table S1.** Oil Red O (ORO) staining on 10 μm longitudinal cryostatic sections of *M. galloprovincialis* digestive gland. The pictures represent the content of neutral lipids in PCTS exposed for 72 h to CTRL (A, C, E) and FFB (B, D, F); and to CTRL (G, I, M) and CAU (H, L, N). CTRL, control; FFB, fenofibrate; CAU, caulerpin.

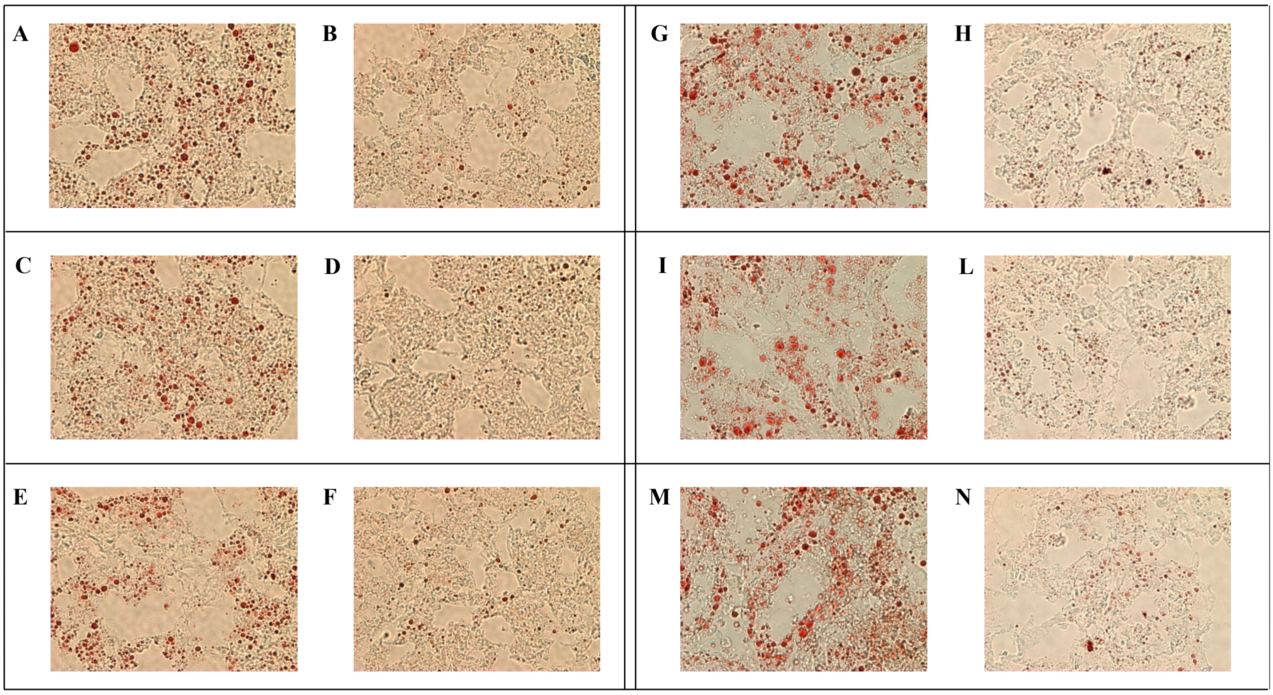

**Table S2.** Schmrol reaction staining on 10 µm longitudinal cryostatic sections of *M. galloprovincialis* digestive gland. The pictures represent the content of lipofuscin in PCTS exposed for 72 h to CTRL (A, C, E) and FFB (B, D, F); and to CTRL (G, I, M) and CAU (H, L, N). CTRL, control; FFB, fenofibrate; CAU, caulerpin.

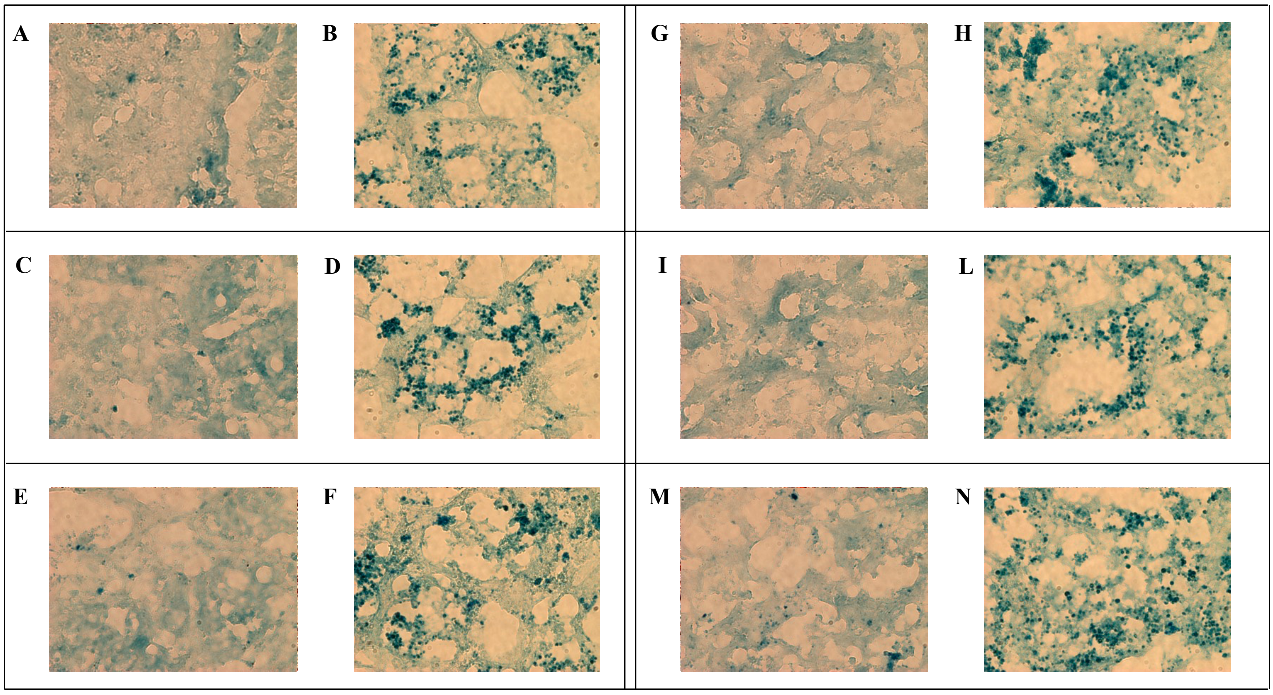

**Table S3.** Sequences of forward and reverse primers and annealing temperatures used for qPCR analysis, amplicon sizes and accession numbers of genes.

| Gene | Primer sequences | Annealing T | Amplicon size | Accession n. |
|---|---|---|---|---|
| *cat* | F: CGACCAGAGACAACCCACC[1]<br>R: GCAGTAGTATGCCTGTCCATCC[1] | 55°C | 132 bp | AY743716 |
| *gst-pi* | F: TCCAGTTAGAGGCCGAGCTGA[2]<br>R: CTGCACCAGTTGGAAACCGTC[2] | 55°C | 172 bp | AF527010 |
| *hsp70* | F: GGTGGTGAAGACTTTGACAACAG [3]<br>R: CTAGTTTGGCATCGCGTAGAGC[3] | 62°C | 295 bp | AY861684 |
| *Cu/Zn-sod* | F: AGCCAATGCAGAGGGAAAAGCAGA[4]<br>R: CCACAAGCCAGACGACCCCC[4] | 65°C | 177 bp | FM177867 |
| *acox1* | F: ACAGTCGTGCAAAACAGGGAC[5]<br>R: CTGCTGCTTCAACCAACCTGG[5] | 62°C | 153 bp | EF525542 |
| *abcb1* | F: CACCATAGCCGAGAACATCC[6]<br>R: CTCCACGCTCTCCAACTAG[6] | 56°C | 140 bp | FM999809 |
| *cyp3A1* | F: TGAACTCGCAAAAAGAACCA[7]<br>R: GGAACACTGGAGCCTTGAAC[7] | 55°C | 80 bp | AY566247 |
